# The importance of full participation: lessons from a national case–control study

**DOI:** 10.1038/sj.bjc.6600092

**Published:** 2002-02-01

**Authors:** United Kingdom Childhood Cancer Study Investigators (writing committee: GR Law, AG Smith, E Roman)

**Affiliations:** Leukaemia Research Fund, Centre for Clinical Epidemiology, 30 Hyde Terrace, University of Leeds, Leeds LS2 9LN, UK

**Keywords:** childhood cancer, aetiology, census, participation, case-control

## Abstract

Differential participation between cases and controls can lead to biased estimates of risk. However, the effects of participation are often ignored. We report a detailed analysis of locations of residence for participants and non-participants in a large, national case–control study of childhood cancer in Great Britain, using the 1991 census. The initial selection of 7669 controls, taken from lists of those registered with a General Practitioner, was representative of the British population in respect to an areal-based index of material deprivation. However, parents of controls agreeing to participate were living in more affluent areas than initially selected controls and their matched 3838 cases. The three components of the deprivation index, persons unemployed, households not owning a car or their home were similarly associated with participation. Other census characteristics, such as proportion of flat dwellers and centrally heated households were also associated with control participation. Population density of the local area was not different between participating controls and their matched cases. However, initially selected controls lived in more urban areas than their cases. Such differences are not unique to this study, as they are an inevitable consequence of incomplete participation. The implications of these differences are discussed, in relation to the difficulty this imposes in the interpretation of studies of disease aetiology.

*British Journal of Cancer* (2002) **86**, 350–355. DOI: 10.1038/sj/bjc/6600092 www.bjcancer.com

© 2002 The Cancer Research Campaign

## 

Voluntary participation in health-related studies is rarely 100%, and there is evidence that in recent years it has been declining (Hartge, 1999). A particular problem for epidemiological research arises when participation is not randomly distributed across study groups. Unfortunately, whether or not an individual agrees to participate in a project is often associated with the health outcome and with the exposure(s) under investigation; invariably this leads to biased estimates of risk (Rothman and Greenland, 1998). The assessment of the impact of differential participation requires the characteristics of those who do not take part to be compared with those who do. In most studies, however, information on non-participators is often sparse or non-existent. Indeed, in some designs, such as those that employ random digit dialling, investigators are not even able to identify non-participants (Wacholder *et al*, 1992).

Previous attempts aimed at exploring this form of selection bias have relied mainly on re-approaching non-participants and asking a restricted number of questions (e.g. Holt *et al*, 1991; Madigan *et al*, 2000; Wrensch *et al*, 2000). However, even when the identities of non-participants are known, practical and ethical considerations often prohibit the use of such methods. The design of the United Kingdom Childhood Cancer Study (UKCCS) provided a valuable opportunity to investigate the potential impact of participation bias (UKCCS Investigators, 2000). The findings, which used small-area census data to investigate differences between participating and non-participating subjects, are reported here.

## MATERIALS AND METHODS

A case–control approach was employed, based around a face-to-face interview of the parents of the index children. Study methods have been described in detail elsewhere (UKCCS Investigators, 2000). The base population was defined as all 0–14 year-olds born and resident in Great Britain (GB), registered with a General Practitioner (GP), without a prior malignancy and not in residential local authority care. Data collection was co-ordinated by 10 regional centres using a common protocol and data collection instruments.

### Case selection

Potentially eligible cases were children diagnosed with a confirmed malignancy or benign tumour of the central nervous system (CNS) within the time frame of the study. All diagnostic groups were included in Scotland between 1991–1994, and 1992–1994 in England and Wales. Case accrual continued in England and Wales for non-Hodgkin's lymphomas and leukaemias during 1995 and leukaemias alone in 1996. Diagnostic confirmation was obtained via several sources: Medical Research Council (MRC) treatment trials, UK Childhood Cancer Study Group (UKCCSG), and histopathology review panels (UKCCS Investigators, 2000).

### Control selection

Two control children were matched on age and sex to each participating case, randomly selected from children registered with the same (former) Family Health Services Authority (England and Wales) or Health Board (Scotland) as their matched case. The first two controls selected were assigned as ‘first-choice’, following successful eligibility checks. In order to restrict the analysis to examining the effects of subject participation and to those with a valid address that could be linked to census, GP permission was obtained for all control families classified as ‘first-choice’. A proportion of the parents of first-choice controls declined to participate and the selection process was repeated until two control families were enrolled and interviewed. The two controls enrolled into the study were assigned as ‘interviewed’, with a proportion of these also assigned as first-choice.

### Linkage to the census of Great Britain

Cases and controls were linked, via the postcode of their residence, to the 1991 census of GB, which was conducted around the time of the commencement of the study (The 1991 census, Crown copyright, ESRC Purchase). Every household has a legal requirement to complete the census form, which contained 25 questions, the content ranging from age and sex of occupants to indicators of affluence such as adult employment and housing conditions. To maintain confidentiality, data are released at an areal level, with the smallest geographical units being indivisible and considered to be homogenous. The smallest units in England and Wales were the 108 336 Enumeration Districts (ED) and in Scotland the 38 084 Output Areas (OA). These can be aggregated into 9527 Electoral Wards (England and Wales) and 1002 Postcode Sectors (Scotland).

The address of the child at the time of diagnosis was used to link the study subjects to the census data. The address was assigned a validated postcode, using an automated system (QuickAddress™) and the Postal Address Books available from Royal Mail. All postcodes were assigned to an ED/OA using a lookup program PC2ED for England and Wales and the Postcode to OA databases for Scotland.

### Census data

The census variables of interest, detailed in Table 1

**Table 1:**
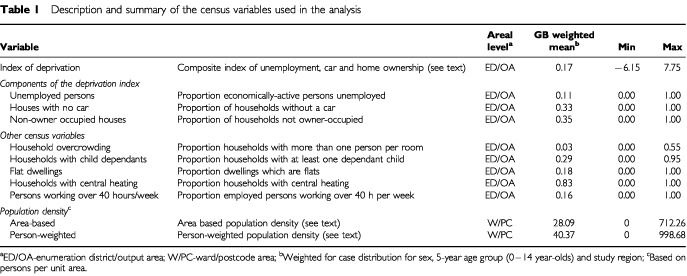
Description and summary of the census variables used in the analysis

, focus on material possessions, employment, household characteristics, and population density. The simple counts provided by the census are given as a proportion of the relevant population (of households or people), as the geographically delimited census regions are not the same size in terms of population or spatial area. The index of deprivation and the assessment of population density are described in more detail below. From Table 2

**Table 2:**
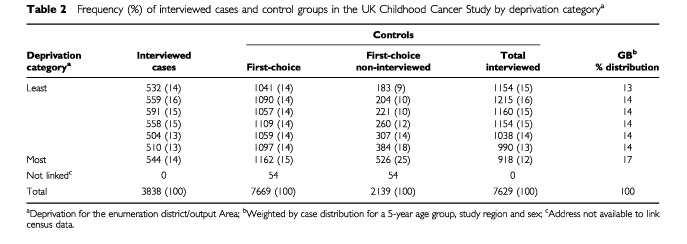
Frequency (%) of interviewed cases and control groups in the UK Childhood Cancer Study by deprivation category^a^

onwards, all variables were divided into seven categories, with wherever possible an equal number of census units for GB in each group. An arithmetic mean for each census measure was calculated by weighting the GB data by the age, sex and regional distribution of cases from the study. The proportions of persons in each of the seven categories from the census, also weighted by the study values were calculated.

#### Deprivation

An index of deprivation was generated for each ED/OA in GB (UKCCS Investigators, 2000). The proportions of unemployed economically active persons (aged 16 and over), households without a car and households not owner-occupied were calculated. Each proportion was transformed to give zero skew with a mean of zero and an overall standard deviation of one. The transformed value for each census unit was calculated and is equivalent to the number of standard deviations from the overall mean. These standard deviations, from the three variables, were summed for each unit and the resulting value is termed the index of deprivation. A high value positive value represents a region with high material deprivation and a high negative value represents an affluent area.

#### Population density

The measures of population density are reported at the electoral ward/postcode sector level. The traditional estimate of population density *d*_*j*_ for each geographical region *j* is given as


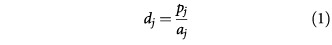


where *p*_*j*_ is the number of persons and *a*_*j*_ the spatial area. This will be referred to as the ‘area-based’ population density and is expressed as persons per hectare. However, this does not necessarily reflect the density at which the ‘average’ person lives, but more closely reflects the land use and type of region. A more useful measure for the density at which an average person lives is termed the ‘person-weighted’ population density (Dorling and Atkins, 1995). This is defined as


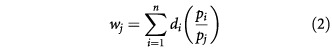


where region *j* is divided into *n* smaller regions *i*, *d*_*i*_ is the area-based population density and *p*_*i*_ the number of persons for the smaller region. For the purposes of this analysis, this may be regarded as a population-weighted sum of the area-based population densities for the ED/OA in each electoral ward/postcode sector. In order to provide a more intuitive assessment of urban-rural status both measures of population density for all persons were categorized as urban (more than 25 persons per hectare), rural (less than 1.5 persons per hectare) or suburban (in between).

### Statistical methods

The risk associated with each explanatory exposure measure is presented as odds ratios from a logistic regression model. Adjustment was made for single year of age, sex and study region as a representation of the matching variables (UKCCS Investigators, 1999). Two comparison groups, not mutually exclusive, were used: the first-choice controls and the interviewed controls.

## RESULTS

Of the 4433 cases identified as eligible, permission to approach 4306 (97%) was obtained from the treating consultant, and 3838 (87%) agreed to participate (Table 2). Case interview rates varied by diagnostic group, from 93% for acute lymphoblastic leukaemia to 82% for the CNS tumours. Of the 7669 matched controls eligible for the study, 5530 (72%) agreed to participate; a lower proportion than for the cases. The proportion for control participation was broadly similar across the different diagnostic groups.

The distribution of interviewed cases and interviewed and first-choice controls by deprivation category is shown in Table 2. The frequency distribution of persons recorded on the census within each of the seven deprivation categories in GB, weighted for the case distribution, is shown on the far right-hand side. The first-choice control group was similarly distributed to that for the whole of GB, with a slightly lower proportion of controls from the most deprived group. The non-interviewed first-choice controls show a skew towards the more deprived groups, with 25% of those refusing to be interviewed coming from the most deprived seventh (where 15% would be expected). This resulted in the interviewed control group having fewer controls from the more deprived areas, with corresponding excesses in the more affluent areas. The distribution for interviewed cases was more similar to the first-choice controls, and to GB as a whole, than to the interviewed controls.

Table 3

**Table 3:**
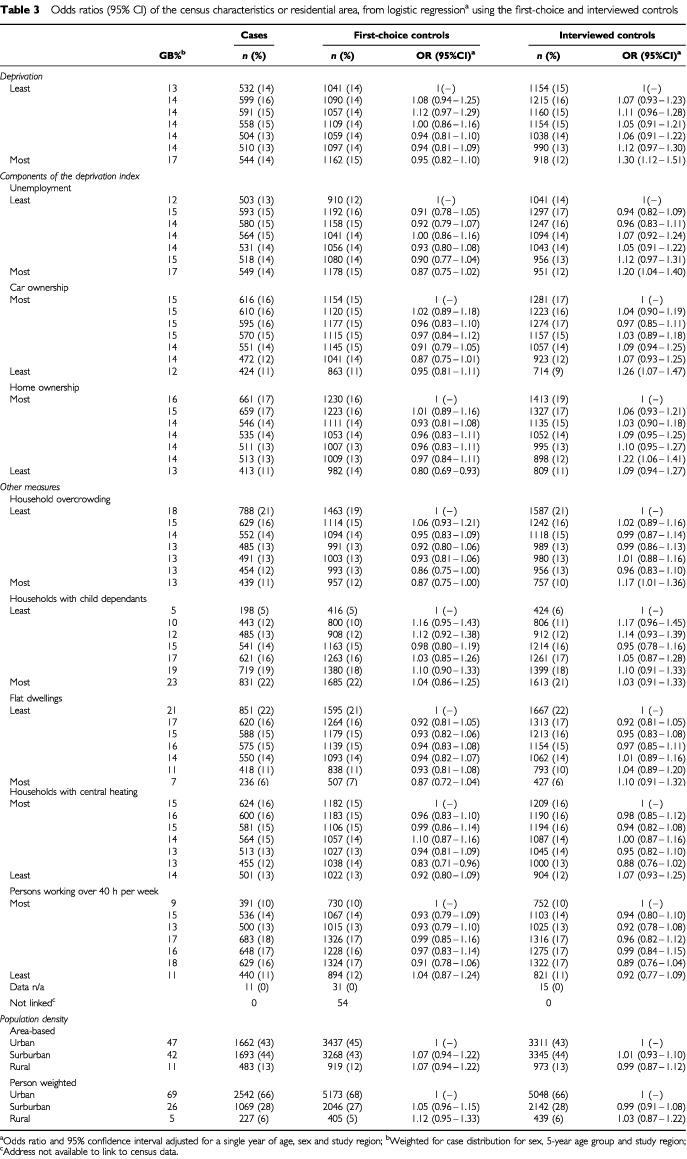
Odds ratios (95% CI) of the census characteristics or residential area, from logistic regression^a^ using the first-choice and interviewed controls

shows the distribution of cases and controls for all chosen census characteristics. The results from a logistic model, adjusting for age, sex and study region are presented, comparing cases to interviewed controls (right hand columns) and to the first-choice controls (central columns). Overall, the trends for the two comparison groups – which are not mutually exclusive – appear to convey conflicting messages. Comparing cases and first-choice controls with respect to deprivation and its component indices, there is little evidence of any consistent associations, with the possible exception of home ownership. However, comparison of interviewed controls with cases suggests elevated risks for childhood cancer associated with increasing material deprivation, unemployment, decreased car ownership and home ownership.

There were no discernable associations between cancer in children and the proportion of houses with child dependants. A non-significant protective effect of proportion of flat dwellers in an area was shown for the first-choice controls. The patterns of association are less clear for the measure of proportion of households with central heating and persons working more than 40 h per week, but the trends are contradictory between the two comparison groups.

Comparison with the interviewed control group, suggested no association between population density and childhood cancer. When making comparison with first-choice controls, no risk estimates for population density were observed to be significantly different from one. However, the point estimates suggested association between cancer and increasing rurality, as measured by both population density methods.

## DISCUSSION

Issues of participation are significant for all studies of disease aetiology that rely on individual compliance. The issue of non-participation, and consequential bias that may be introduced, is critically important in case–control studies that rely on personal contact to assess environmental experiences and exposures. Our findings indicate that in such studies, the profiling of non-participants may be as important as that of participants. In the study described here, the involvement of parents required active participation, as information was primarily collected through a face-to-face interview (UKCCS Investigators, 2000). As with all interview-based case-control studies, however, whilst the motivation of affected families to participate was strong, the motivation of those who were unaffected is less clear.

Comparisons of census data for cases and first-choice controls provided results that were free from participation bias: the findings suggesting that, on average, case families tended to live in areas that were more affluent than those of control families. In contrast, comparison with participating control families suggested that, on average, case families tended to live in areas that were less affluent than those of control families. This observation is consistent with other reports suggesting that participants often belong to a higher socio-economic groups than non-participants as measured by housing tenure, income, level of education and occupation (Holt *et al*, 1991; Hatch *et al*, 1998; Madigan *et al*, 2000; Wrensch *et al*, 2000).

Measures of material deprivation are often closely associated with possible aetiological factors such as smoking, occupation, and previous illness history. Indeed, in many epidemiological studies it is virtually impossible to identify potentially harmful exposures that are not – either directly or indirectly – related to measures of social class, deprivation or affluence. Further, somewhat surprisingly, the treatment of bias as a real confounder (risk factor) rarely impacts on the magnitude of risk estimates (e.g. UKCCS Investigators, 1999; 2001). The challenge is to disentangle the artifactual consequences of participation bias from genuine aetiological factors. Some researchers have suggested using the variable most closely related to participation as a confounder to ‘adjust’ for participation. Such adjustments are, however, only appropriate when the antecedents of both exposure and disease, or their joint distributions, are available for the entire study population (Greenland, 1998). As in the majority of studies, neither of these were available for the UKCCS, where individual reasons for participation could only be indirectly related with the deprivation index.

As expected, our findings show that the primary care sampling frame provided control families that were broadly representative of the general GB population, where the majority of people register with a GP in the area in which they live – access to NHS medical care requiring registration which covers approximately 98% of the population (RCGP, 1987). The UKCCS sampling frame compares favourably with other methods of control selection. For example, random digit dialling, a method commonly employed in the USA and Canada (Robison and Daigle, 1984), prohibits collection from homes without a telephone, those not at home when telephoned, and those who answer but who either refuse to answer any questions or deliberately lie about their families eligibility.

In conclusion, our findings confirm that differential participation is a potentially major source of bias in case–control studies that estimate risks on the basis of information reported from respondents alone. Studies that ignore this source of bias may produce misleading results. There is a clear need to address this issue in terms of study design, and in the application of appropriate statistical methods to try to overcome this bias.

## APPENDIX 1

*Writing Committee* – GR Law, AG Smith, E Roman

*Management Committee* – KK Cheng, Central Region; N Day, East Anglia Region; R Cartwright, A Craft, North East Region; JM Birch, OB Eden, North West Region; PA McKinney, Scotland; J Peto, South East Region; V Beral, E Roman, South Midlands Region; P Elwood, South Wales Region; FE Alexander, South West Region; CED Chilvers, Trent Region; R Doll, Epidemiological Studies Unit, University of Oxford; GM Taylor, Immunogenetics Laboratory, University of Manchester, Manchester; M Greaves, Leukaemia Research Fund Centre, Institute of Cancer Research; D Goodhead, Radiation and Genome Stability Unit, Medical Research Council, Harwell; FA Fry, National Radiological Protection Board; G Adams, UK Co-ordinating Committee for Cancer Research.

*Regional Investigators* – KK Cheng, E Gilman, Central Region; N Day, J Skinner, D Williams, East Anglia Region; R Cartwright, A Craft, North East Region; JM Birch, OB Eden, North West Region; PA McKinney, Scotland; J Deacon, J Peto, South East Region; V Beral, E Roman, South Midlands Region; P Elwood, South Wales Region; FE Alexander, M Mott, South West Region; CED Chilvers, K Muir, Trent Region.

*Data Processing Group* – GR Law, J Simpson, E Roman.

A complete list of investigators is given in: The United Kingdom Childhood Cancer Study: objectives, materials, and methods. *Br J Cancer* 2000; **82:** 1073–1102.
